# Feasibility of Robotic-Assisted Mitral Ring Annuloplasty Using the Continuous Wrapping Suture Technique: A Single-Centre Experience

**DOI:** 10.1093/icvts/ivaf223

**Published:** 2025-10-07

**Authors:** Kazuki Noda, Yosuke Takahashi, Akimasa Morisaki, Kenta Nishiya, Goki Inno, Takumi Kawase, Yukihiro Nishimoto, Munehide Nagao, Ryo Nangoya, Toshihiko Shibata

**Affiliations:** Department of Cardiovascular Surgery, Osaka Metropolitan University, Osaka 545-8585, Japan; Department of Cardiovascular Surgery, Osaka Metropolitan University, Osaka 545-8585, Japan; Department of Cardiovascular Surgery, Osaka Metropolitan University, Osaka 545-8585, Japan; Department of Cardiovascular Surgery, Osaka Metropolitan University, Osaka 545-8585, Japan; Department of Cardiovascular Surgery, Osaka Metropolitan University, Osaka 545-8585, Japan; Department of Cardiovascular Surgery, Osaka Metropolitan University, Osaka 545-8585, Japan; Department of Cardiovascular Surgery, Osaka Metropolitan University, Osaka 545-8585, Japan; Department of Cardiovascular Surgery, Osaka Metropolitan University, Osaka 545-8585, Japan; Department of Cardiovascular Surgery, Osaka Metropolitan University, Osaka 545-8585, Japan; Department of Cardiovascular Surgery, Osaka Metropolitan University, Osaka 545-8585, Japan

**Keywords:** mitral valve annuloplasty, ring annuloplasty, continuous wrapping suture

## Abstract

**Objectives:**

Robotic-assisted mitral ring annuloplasty is safe and effective; however, the use of conventional interrupted suture may prolong operative time. This study retrospectively evaluated the safety and feasibility of continuous wrapping suture for annuloplasty in robotic-assisted mitral valve (MV) repair.

**Methods:**

This study included 581 patients who underwent MV repair with annuloplasty ring replacement at our institution between 2010 and 2023. Among the 581 patients, 168 who underwent MV repair with the continuous wrapping suture technique were included in the main analysis. In the continuous wrapping suture technique, the ring was fixed at both the fibrous trigones of the anterior leaflet and was continuously sutured to wrap it circumferentially. The primary outcomes were perioperative procedure-related complications and ring dehiscence during the observation period.

**Results:**

The median follow-up duration was 2.7 years. There were no annuloplasty-related complications, including left circumflex artery (LCX) injury and prosthetic ring dehiscence. The 1-year and 3-year postoperative recurrence rates of moderate or severe mitral regurgitation were 1.8% and 2.2%, respectively. Moreover, in the whole 581 patients who underwent mitral ring annuloplasty, multivariable analysis confirmed that the wrapping suture technique was significantly associated with a reduction in the 1-year postoperative mean MV pressure gradient (mean ratio 0.802; 95% CI, 0.728-0.884; *P* < .001).

**Conclusions:**

The wrapping suture technique for robotic-assisted MV repair with ring annuloplasty is safe and effective, with no observed cases of LCX injury or ring dehiscence, and may serve as a reasonable alternative in anatomically challenging cases.

## Graphical Abstract

**Figure:**
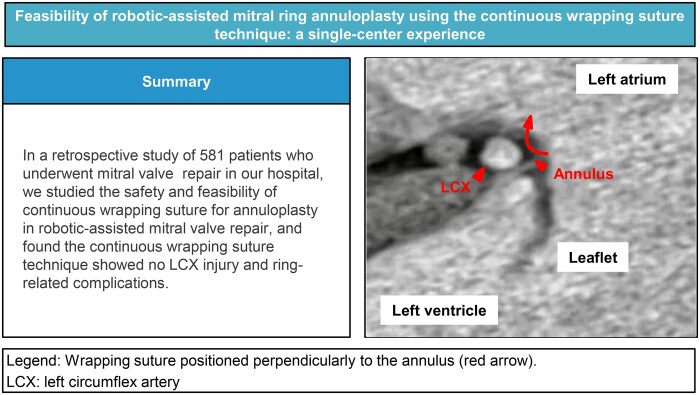

## INTRODUCTION

Robotic-assisted mitral valve (MV) repair has emerged as a safe and effective method for treating MV pathology.^1–3^ Several studies have also reported its feasibility and safety for more complex mitral lesions, and concomitant procedures have been reported in several studies.^4^^,^^5^ To achieve complex valve repair, we have modified our ring annuloplasty technique by utilizing the advantages of robotic-assisted surgery, rendering the procedure more streamlined and reproducible.

Rings are typically secured with multiple interrupted sutures, each anchored with individual mattress sutures. This process prolongs the operative time in robotic-assisted MV repair owing to the need for suture material exchange, time-consuming knot tying, and reliance on assistance from bedside surgeon.^6^

In 2018, we initiated robotic-assisted MV repair with annuloplasty using the continuous wrapping suture technique^2^^,^^7^ as an alternative for the conventional interrupted suture technique owing to the latter’s technical complexity under limited visibility. The conventional technique involves passing the suture from the left atrium to the left ventricle and then back to the left atrium. Meanwhile, the wrapping suture technique follows a perpendicular approach to the annulus, in which the suture passes from the annulus to the left atrium. However, the technical advantages and complications of continuous wrapping suture for annuloplasty have not yet been fully elucidated.

This study aimed to retrospectively investigate the early and mid-term outcomes of robotic-assisted MV repair with annuloplasty using the continuous wrapping suture and assess the safety and feasibility of this technique.

## PATIENTS AND METHODS

### Ethics statement

This study did not involve the collection or storage of patient data or biological materials for multiple or indefinite use, and no third-party managed databases or biobanks were utilized. Before surgery, written informed consent for the procedure and the use of data were obtained from the patients. Data collection, analysis, and reporting were approved by the Osaka Metropolitan University Institutional Review Board (Reference number 2024-015).

### Study cohort and data collection

**Figure 1.**
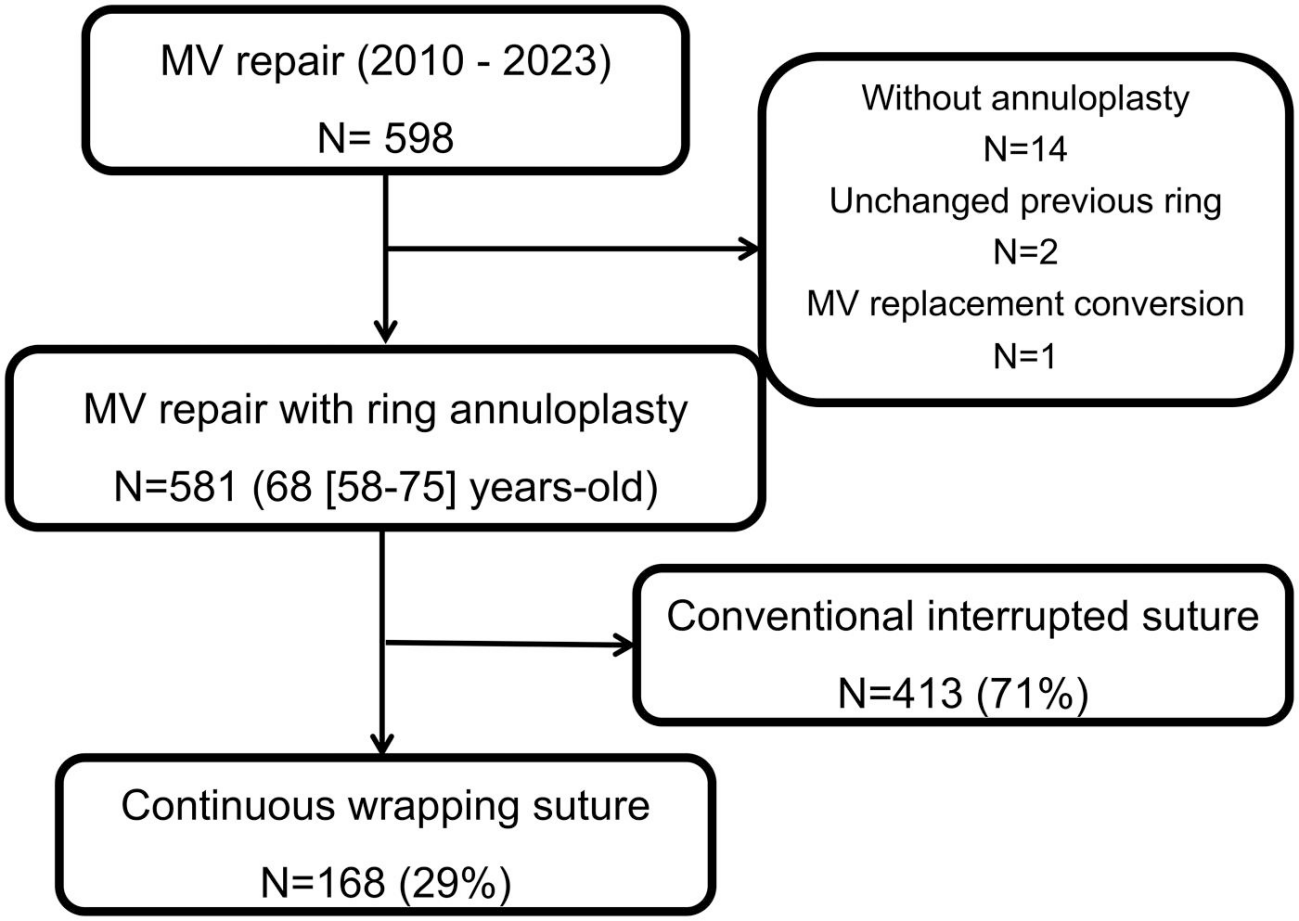
Flow Chart of Patient Selection.

The institutional surgical database included 598 patients who underwent MV repair in Osaka Metropolitan University Hospital between 2010 and 2023. Of them, 14 who underwent MV repair without annuloplasty for primary mitral regurgitation (MR) due to infective endocarditis or cardiac tumour, 2 who underwent MV re-repair without removal of the previous ring, and 1 who underwent intraoperative conversion of MV replacement following annuloplasty were excluded from the study. Among the 581 patients, 168 who underwent MV repair with the continuous wrapping suture technique were included in the main analysis. The continuous wrapping suture technique was consistently performed by a single surgeon throughout the study period, ensuring procedural uniformity. The remaining 413 patients who underwent the conventional interrupted suture technique by multiple surgeons were not included in the primary analysis but were analysed separately in subgroup analysis and described in the baseline characteristics table (**Figure 1**).

### Echocardiography and surgical indication

Transthoracic echocardiography was routinely performed within 1 week preoperatively, 1 week postoperatively, and annually thereafter. The regurgitation severity was classified as none, trivial, mild, mild to moderate, moderate, and severe using colour flow Doppler data. The MR type was determined according to the Carpentier classification.^8^ A minimally invasive approach or robotic-assisted MV repair was indicated for clinically relevant MR, except for cases with severe lung disease, moderate aortic regurgitation, severe left ventricular dysfunction, and a previous history of cardiac surgery. As part of the preoperative assessment, three-dimensional cardiac computed tomography (3D-CT) was routinely performed to evaluate the anatomical relationship between the left circumflex artery (LCX) and the posterior mitral annulus. The surgical robot used in this study was the da Vinci Surgical System Si and Xi (Intuitive Surgical Inc., Sunnyvale, CA, United States). The institutional heart team, which consisted of cardiologists, radiologists, and surgeons, made the final decision with primary reference to published guidelines.^9^^,^^10^

### Procedure

The MV was observed via a right-sided left atrial approach through a right mini-thoracotomy in all cases. All the patients in this study underwent MV repair with annuloplasty. Chordal reconstruction with or without the use of the loop technique^11^ was performed if leaflet prolapse occurred. Furthermore, autologous pericardial patch augmentation of the posterior leaflet was performed if a large gap between the anterior and posterior leaflets due to severe posterior leaflet tethering was observed. Conversely, simple height reduction was performed at the surgeon’s discretion^12^ if there was a high risk of systolic anterior motion with the redundant posterior leaflet. After intercommissural distance and anterior MV sizing, a prosthetic ring was implanted.

### Ring annuloplasty

**Figure 2.**
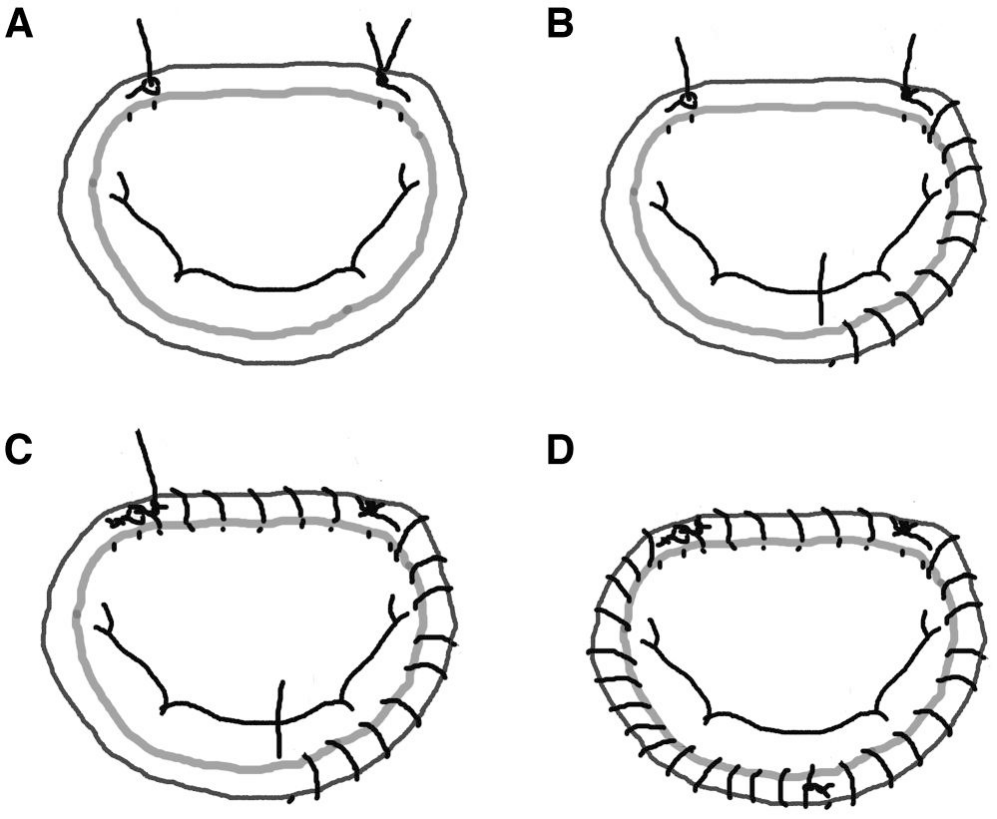
Schemas of the Mitral Annuloplasty with a Full Ring Using the Continuous Wrapping Suture. First, a Prosthesis Ring Is Attached with CV4 Polytetrafluoroethylene Sutures at Both Fibrous Trigones of the Anterior Leaflet (A). Next, the Ring Is Attached Clockwise from the Medial Fibrous Trigone to the Midportion of the Posterior Leaflet (B). The Sutures Are Then Continued along the Anterior Leaflet (C). Finally, the Ring Is Attached Counterclockwise from the Lateral Fibrous Trigone Back to the Midportion of the Posterior Leaflet (D).

In the continuous wrapping suture technique, a prosthetic ring was attached to the mitral annulus with two CV4 polytetrafluoroethylene sutures (GORE-TEX; W.L. Gore and Associates Inc., Newark, DE, United States). The sutures were placed starting at both the fibrous trigones of the anterior leaflet. They were initially applied clockwise from the medial fibrous trigone to the midportion of the posterior leaflet while adjusting the placement of the ring to ensure uniform annular reduction. For a full ring, the sutures were continued along the anterior leaflet. Finally, the sutures were applied counterclockwise from the lateral fibrous trigone back to the midportion of the posterior leaflet. The procedure for continuous wrapping suturing is shown in **Figure 2A-D** and **Supplementary Video 1**.

In the conventional interrupted suture technique, individual sutures were placed circumferentially around the mitral annulus with 2-0 polyester sutures. Hand-tying was performed using a knot pusher in most cases to ensure secure fixation of the annuloplasty ring. In more recent cases, however, the COR-KNOT device (LSI Solutions Inc., Victor, NY, United States) has also been used as an alternative for suture fastening.

### Definitions and clinical follow-up

The primary outcome was intra- or postoperative procedure-related complications, including LCX injury and prosthesis ring dehiscence. Other outcomes of interest were mortality, major complications, an unscheduled heart failure admission, MV reintervention, change in MR severity following surgery, and suture time required for only ring annuloplasty using the wrapping suture technique. The diagnosis of postoperative recurrent heart failure was based on clinical symptoms, physical signs, or radiological evidence of pulmonary congestion. Suture time was defined as the duration from suture placement at the fibrous trigone to knot tying. Postoperative follow-up was conducted in outpatient clinics and via telephone survey and was completed by all patients with a median duration of 2.7 (interquartile range [IQR], 1.5-3.9) years. Postoperative follow-up with echocardiography at 1 year was conducted in 97% of the patients.

### Statistical analysis

Categorical data were expressed as frequencies and proportions and compared using the chi-squared test or Fisher’s exact test as appropriate. Meanwhile, continuous data were expressed as median with IQRs; unpaired data were compared using the Mann-Whitney *U* test, whereas paired data were compared using the Wilcoxon signed-rank test. Statistical significance was defined as 2-tailed *P* < .05 and standardized mean difference (SMD) > 0.10. No adjustments were made for multiple comparisons. Missing data were limited to a few postoperative echocardiographic findings. These data were treated as “not available” and excluded from the relevant analyses. The outcomes were assessed using linear, logistic, or Cox proportional-hazards regression to adjust for confounding factors. Robust standard errors, including the sandwich estimator, were applied to account for potential violations of model assumptions. All *P*-values may not be interpreted as confirmatory but descriptive. The R software version 4.3.1 (The R Foundation for Statistical Computing, Vienna, Austria) was used to conduct statistical analyses.

## RESULTS

### Patient background

The patients’ background characteristics are presented in **Table 1**. The median age was 62 (IQR, 53-71) years. Preoperative echocardiography revealed that the MR severity was moderate or severe in 165 (98.2%) patients. Furthermore, a high proportion of patients had Carpentier type 2 MR (92.3%). A total of 121 (72%) patients underwent annuloplasty using a full ring, whereas 119 (70.8%) underwent annuloplasty using a rigid or semi-rigid type of ring.

**Table 1.** Patient Characteristics

Preoperative variables	Interrupted suture (*n* = 413)	Wrapping suture (*n* = 168)	SMD
Age (years)	70 (60, 76)	62 (53, 71)	0.452
BSA (m^2^)	1.59 (1.44, 1.72)	1.65 (1.52, 1.81)	0.059
Male	232 (56.0%)	104 (61.9%)	0.117
NYHA class 3 or 4	71 (17.2%)	3 (1.8%)	0.545
Atrial fibrillation	164 (39.7%)	37 (22%)	0.390
Hypertension	234 (56.7%)	84 (50.0%)	0.134
Dyslipidaemia	121 (29.3%)	38 (22.6%)	0.153
Diabetes mellitus	60 (14.5%)	12 (7.1%)	0.239
Old cerebral infarction	38 (9.2%)	9 (5.4%)	0.148
Coronary artery disease	80 (19.4%)	16 (9.5%)	0.283
Low-EF (EF <30%)	13 (3.2%)	0 (0%)	0.256
Obstructive ventilatory defect (FEV1.0% <70%)	77 (18.6%)	31 (18.5%)	0.005
Renal dysfunction (Cre >1.5 mg/dl)	53 (12.8%)	7 (4.2%)	0.315
Liver cirrhosis	5 (1.2%)	1 (0.6%)	0.065
Previous open-heart surgery	16 (3.9%)	0 (0%)	0.284
Previous mitral valve procedure	1 (0.2%)	0 (0%)	0.070
Previous pacemaker implantation	7 (1.7%)	0 (0%)	0.186
Preoperative echocardiography findings			
MR grade moderate or severe	337 (81.6%)	165 (98.2%)	0.574
Carpentier classification of MR			0.848
Type 1	143 (34.6%)	12 (7.1%)	
Type 2	247 (59.8%)	155 (92.3%)	
Type 3a	1 (0.2%)	1 (0.6%)	
Type 3b	22 (5.3%)	0 (0%)	
Ring type			
Full ring	380 (92%)	121 (72%)	0.539
Rigid or semi-rigid ring	401 (97.1%)	119 (70.8%)	0.766
Ring size (mm)	28 (28, 30)	30 (29, 32)	0.868

Continuous variables are presented as median (IQR), whereas categorical variables are presented as number (%).

Abbreviations: BSA, body surface area; Cre, serum creatinine level; EF, ejection fraction; FEV, forced expiratory volume; MR, mitral regurgitation; NYHA, New York Heart Association; SMS, standardized mean difference.

### Procedural data

Details of the surgery and concomitant procedures are summarized in **Table 2**. The robotic-assisted approach was applied in all patients with the wrapping suture technique. Most of all were performed with the additional MV procedure, not only mitral ring annuloplasty.

**Table 2.** Perioperative Outcomes

Variables	Interrupted suture (*n* = 413)	Wrapping suture (*n* = 168)	SMD
Operative details			
Operative approach			
Median sternotomy	346 (83.8%)	0 (0%)	3.214
Robotic	10 (2.4%)	168 (100%)	8.978
MICS	57 (13.8%)	0 (0%)	0.566
Additional mitral valve procedure	314 (76.0%)	159 (94.6%)	0.545
Loop technique or chordal reconstruction	231 (55.9%)	158 (94.0%)	0.980
Resection and suture	20 (4.8%)	1 (0.6%)	0.263
Leaflet plication	207 (50.1%)	71 (42.3%)	0.158
Height reduction	18 (4.4%)	23 (13.7%)	0.330
Posterior leaflet extension or patch reconstruction	19 (4.6%)	1 (0.6%)	0.254
Concomitant procedures			
Maze procedure	72 (17.4%)	29 (17.3%)	0.005
Tricuspid valve repair or replacement	172 (41.6%)	33 (19.6%)	0.491
CABG	58 (14.0%)	0 (0%)	0.572
Aortic valve repair or replacement	100 (24.2%)	0 (0%)	0.799
Graft replacement	27 (6.5%)	0 (0%)	0.374
Operation time (min)	328 (272, 418)	296 (261, 344)	0.450
Cardiac arrest time (min)	159 (130, 188)	134 (107, 165)	0.576
Second arrest	48 (11.6%)	6 (3.6%)	0.307
Transfusion	366 (88.6%)	85 (50.6%)	0.908

Continuous variables are presented as median (IQR), whereas categorical variables are presented as number (%).

Abbreviations: CABG, coronary artery bypass grafting; MICS, minimally invasive cardiac surgery; SMS, standardized mean difference.

### Postoperative outcomes

No patient experienced in-hospital death. Notably, as shown in **Table 3**, there were no annuloplasty-related complications, including LCX injury and prosthetic ring dehiscence.

**Table 3.** Intra- and Postoperative Complications

Variables	Wrapping suture (*n* = 168)
In-hospital death	0 (0%)
ICU and HCU day (days)	2 (2, 2)
Hospital day (days)	10 (9, 12)
Inoperative LCX injury	0 (0%)
Other coronary events	0 (0%)
Re-sternotomy or conversion	2 (1.2%)
Mediastinitis	0 (0%)
Aortic dissection	0 (0%)
Pacemaker implantation	0 (0%)
Cerebrovascular accident	1 (0.6%)
Acute renal failure requiring dialysis	0 (0%)
The 1-year postoperative echocardiography findings	
MR grade moderate or severe	3 (1.8%)
Mean MV pressure gradient (mmHg)	2 (2, 3)
Long-term outcomes	
Long-term mortality	2 (1.2%)
Admission for cardiac failure	4 (2.4%)
Ring dehiscence	0 (0%)
Mitral valve reintervention	1 (0.6%)

Continuous variables are presented as median (IQR), whereas categorical variables are presented as number (%).

Abbreviations: HCU, high care unit; ICU, intensive care unit; LCX, left circumflex artery; MR, mitral regurgitation; MV, mitral valve.

**Figure 3.**
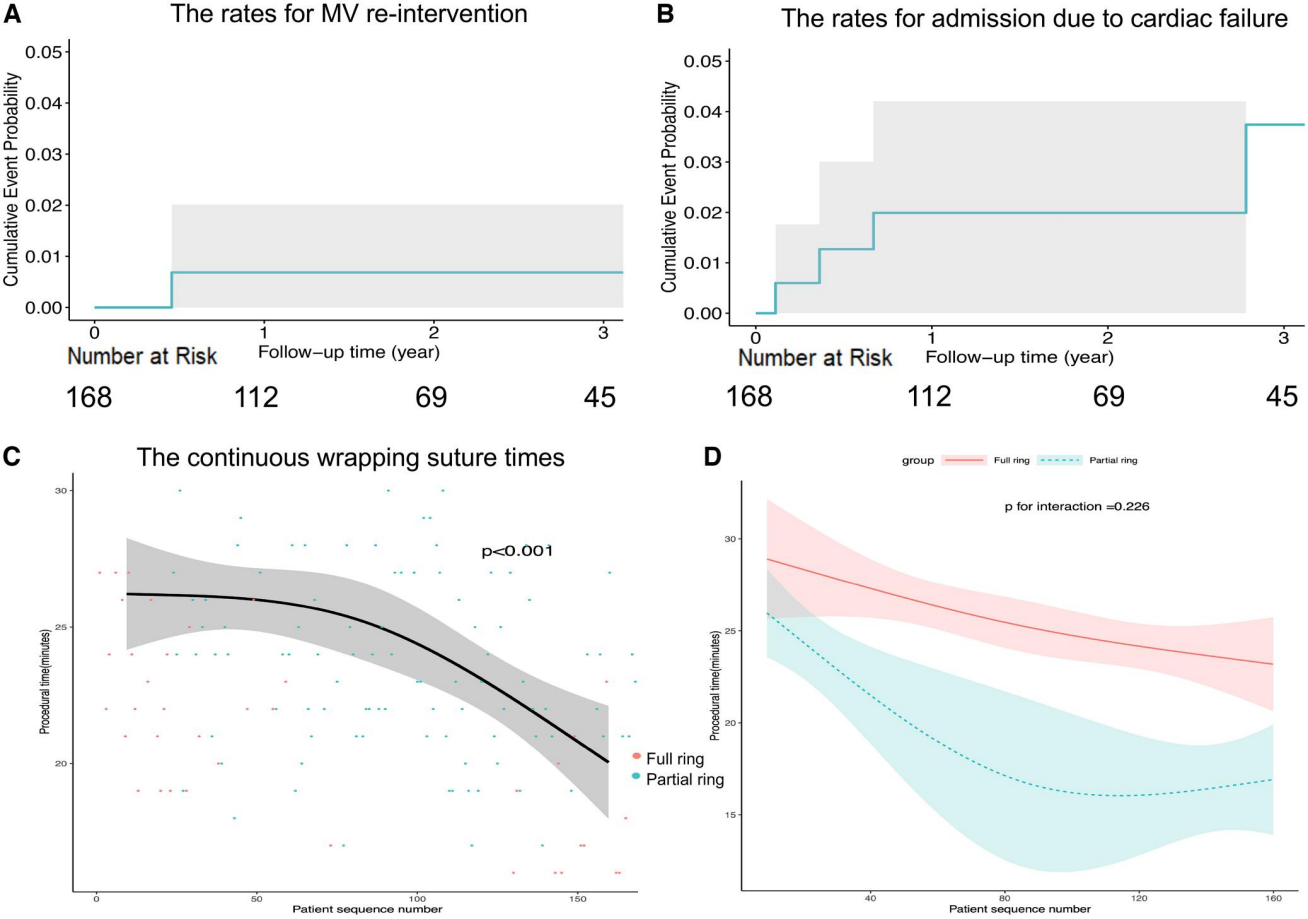
Cumulative Event Rates for Mitral Valve re-Intervention (A) and Hospital Admission Due to Cardiac Failure (B). The Learning Curve and Scatter Plot of the Wrapping Suture Times (C) and the Learning Curve of the Procedural Times, Stratified by Full or Partial Ring Usage (D).

The 1- and 3-year cumulative probability of MV reintervention were 0.7% (95% CI, 0%-2%) and 0.7% (95% CI, 0%-2%), respectively (**Figure 3A**). Moreover, the respective 1- and 3-year cumulative probability of admission for cardiac failure were 2.0% (95% CI, 0%-4.2%) and 3.7% (95% CI, 0%-7.7%), respectively (**Figure 3B**). The 1-year and 3-year postoperative recurrence rates of moderate or severe MR were 1.8% and 2.2%, respectively.

### Minimal learning curve in the wrapping suture technique

The median suture time required for only the ring annuloplasty procedure using the wrapping suture technique was 23 (IQR, 21-27) min. The learning curve and scatter plot of the procedural time are presented in **Figure 3C**. The procedural time significantly decreased with each successive case (*P* < .001), particularly after surpassing 50 cases. The learning curve of the procedural time, stratified by ring type (full or partial), is illustrated in **Figure 3D**. Similarly, the procedural time significantly decreased, particularly for the partial ring group, whereas the interaction between the full or partial ring groups was not statistically significant (*P* = .226).

### Factors affecting the 1-year postoperative MV function in the subgroup analysis

In the whole 581 patients who underwent mitral ring annuloplasty, multivariable logistic regression analysis, with adjustment for MV repair procedures and the ring type, revealed that the use of the wrapping suture technique was not a significant factor for predicting the 1-year recurrence of moderate or severe MR (OR 0.528; 95% CI, 0.146-1.90; *P* = .328) (**Table 4**). In contrast, the posterior leaflet extension or patch reconstruction was identified as an independent risk factor for MR recurrence (OR 6.41; 95% CI, 1.62-25.3; *P* = .008) (**Supplementary Table S1**). Moreover, the wrapping suture technique was confirmed to be significantly associated with a reduction in the 1-year postoperative mean MV pressure gradient in the same model (mean ratio 0.802; 95% CI, 0.728-0.884; *P* < .001) (**Table 4**). In addition, the pressure gradient decreased with increasing ring size (mean ratio 0.948; 95% CI, 0.933-0.964; *P* < .001), suggesting a size-dependent reduction effect (**Supplementary Table S2**).

**Table 4.** Univariate and Multivariate Regression Analysis on Outcomes in the Whole Patients Who Underwent Mitral Ring Annuloplasty

Outcomes	Wrapping suture	
	Univariate	Multivariate^a^
1-year postoperative MR grade moderate or severe Odds ratio (95% CI) *P*-value	0.449 (0.127-1.58) .213	0.528 (0.146-1.90) .328
1-year postoperative mean MV pressure gradient Mean ratio (95% CI) *P*-value	0.709 (0.654-0.770) <.001	0.802 (0.728-0.884) <.001

a See adjusted models shown in **Supplementary Tables S1 and S2**.

Abbreviations: MR, mitral regurgitation; MV, mitral valve.

## DISCUSSION

This study evaluated the early and mid-term outcomes of robotic-assisted MV repair with annuloplasty using the continuous wrapping suture technique. The patients who underwent this procedure did not develop related complications or prosthetic ring dehiscence.

We suggested that robotic-assisted mitral ring annuloplasty using the wrapping suture offers several advantages, particularly in robotic surgery. First, this procedure may improve the efficiency of ring annuloplasty by reducing the procedural workload. In particular, the continuous suture technique minimizes the need for frequent suture exchanges and simplifies intraoperative suture handling, which can be particularly advantageous in the limited operative field of robotic surgery. In addition, the wrapping suture technique has cost-saving implications. Interrupted suture often requires time-consuming knot tying, thereby prolonging the aortic cross-clamp time. Although the COR-KNOT device (LSI Solutions Inc., Victor, NY, United States), an automated suture fastening system, can reduce the procedural time and improve efficiency in the interrupted sutures,^13^ its high cost cannot be overlooked. To reduce the knot-tying time, we used a pre-knotted CV4 suture,^14^ which was tied over a nerve hook, and modified it so that only one final knot was required. Therefore, the wrapping suture technique is more cost-effective, making it a reasonable alternative.

There have been reports on the safety and effectiveness of the use of barbed sutures for running sutures, specifically for partial annuloplasty.^3^^,^^15^ As the suturing alternates between the annulus and the ring in the running suture, the suture time is much shorter compared to the wrapping sutures. However, the portion of the suture attached to the ring could not effectively engage the annulus, which may result in suboptimal mitral annulus plication. Furthermore, the application of the same running sutures to a full ring may be challenging. In contrast, although the wrapping suture technique requires adjustment of the suture position where the suture is placed on the prosthetic ring with each stitch, it allows for a more accurate and uniform annuloplasty. In addition, securing the centre of the P2 segment with a temporary suture before plication may facilitate a more uniform plication. The approximately 20% reduction in the postoperative mean MV pressure gradient compared to the interrupted suture is potential evidence of more uniform annuloplasty.

LCX stenosis or occlusion during mitral annuloplasty can occur due to stitching of the mitral annulus, including direct injury and distortion of the artery caused by excessive tissue retraction, particularly from large or deep suture bites.^16^ Therefore, we needed to take care to avoid taking large and deep bites, especially when suturing the lateral side of the mitral annulus. Additionally, approximately 5 shallow stitches are typically placed from the anterolateral commissure to the P1 segment in our cases, which may help reduce the risk of ring dehiscence. To prevent these complications, preoperative 3D-CT is employed to recognize the anatomical relationship between the LCX and the mitral annulus.^17^ By identifying factors such as the close proximity of the LCX and the left coronary artery dominance, which are potential risk factors for LCX injury,^18^ we ensured the safe application of the wrapping suture technique.

In cases with close anatomical coronary annular distance, as illustrated in the central image, passing the suture from the left atrium to the left ventricle and exiting again on the atrial side, which essentially represents parallel needle insertion to the annulus, may increase the risk of LCX injury. The wrapping suture technique, which involves a perpendicular insertion to the annulus, may be advantageous in such anatomically constrained settings. No LCX injury was observed in our cohort, suggesting that this technique may be a viable alternative to conventional approaches.

Mitral prosthetic ring dehiscence is a rare but challenging procedural failure.^19^ Although numerous factors are known to increase the risk of ring dehiscence, this multifactorial complication can generally be categorized into 4 domains: ring characteristics, valve pathology, patient characteristics, and technical factors.^20^ Notably, despite the use of a rigid ring in the majority of cases, ring dehiscence did not occur, highlighting the clinical utility of the wrapping suture technique. However, the annular reduction rate could not be included as a covariate owing to insufficient data on the relationship between the prosthetic ring size and the precise mitral annulus size. A previous laboratory study reported that undersized rings had higher contractile forces on the sutures as they constricted the annulus, potentially increasing the risk of ring dehiscence.^21^ Clinically, we previously showed that the posterior annular plication rate, which was quantified as the ratio of the posterior ring length to the posterior annular length, may serve as an important parameter for ring size selection,^22^ particularly given that annular dilatation may affect the posterior annular side.^23^ These findings suggested that a certain degree of overplication may be tolerated without resulting in ring dehiscence. Further investigation with long-term follow-up is warranted to evaluate the relationship between the prosthetic ring size and the preoperative 3D-CT measurements of the mitral annular diameter, with the aim of identifying patients at higher risk of ring dehiscence.

### Limitations

The present study was a retrospective study conducted in a single centre and included a limited number of patients. Furthermore, there were a few patients in this study who had ischaemic MR, which is a potential risk factor for ring dehiscence^24^ due to aggressive annular downsizing. Moreover, while many patients underwent ring annuloplasty with rigid and full rings, the choice of the prosthetic ring and surgical technique for MV repair was based on each surgeon’s judgement. Finally, not all patients underwent 1-year follow-up, including echocardiography, which is mandatory to confirm the benefits of MV repair.

## CONCLUSIONS

This study demonstrates that the wrapping suture technique for robotic-assisted MV repair with ring annuloplasty is a safe and effective method, as no LCX injury or ring dehiscence was observed during the early- to mid-term follow-up. These findings suggest that the technique may be a reasonable alternative in selected cases. However, a long-term follow-up is required to evaluate the durability of this technique.

## Supplementary Material

ivaf223_Supplementary_Data

## Data Availability

According to applicable data protection laws, the data that served as the basis for this article cannot be disclosed to the general public. Nonetheless, with the ethics committee’s approval, the data will be made available to the relevant author upon reasonable request.
