# Supplementary material for: Feasibility of Robotic-Assisted Mitral Ring Annuloplasty Using the Continuous Wrapping Suture Technique: A Single-Centre Experience
Source: Interdiscip Cardiovasc Thorac Surg. 2025 Oct 7;40(10):ivaf223. doi: 10.1093/icvts/ivaf223 (PMC12513763; doi:10.1093/icvts/ivaf223)
Supplement: ivaf223_Supplementary_Data [file ivaf223_supplementary_data.docx]

Supplementary Table. S1 Risk factors for 1-year postoperative recurrence of moderate or severe MR

| variables | Univariate analysis | | Multivariate analysis | |
| --- | --- | --- | --- | --- |
|  | Odds ratio (95% CI) | p-value | Odds ratio (95% CI) | p-value |
| Wrapping suture | 0.449 (0.127-1.58) | 0.213 | 0.528 (0.146-1.90) | 0.328 |
| Age (years) | 1.01 (0.961-1.05) | 0.765 |  |  |
| Loop technique or chordal reconstruction | 1.33 (0.428-4.16) | 0.620 |  |  |
| Resection and suture | <0.001 | 1 |  |  |
| Leaflet plication | 0.744 (0.279-1.99) | 0.555 |  |  |
| Height reduction | 1.65 (0.364-7.49) | 0.516 |  |  |
| Posterior leaflet extension or patch reconstruction | 7.36 (1.90-28.6) | 0.004 | 6.41 (1.62-25.3) | 0.008 |
| Rigid or semirigid ring | 0.937 (0.209-4.21) | 0.933 |  |  |
| Full ring | 0.807 (0.226-2.88) | 0.741 |  |  |
| Ring size | 0.911 (0.742-1.12) | 0.376 |  |  |

Supplementary Table. S2 1-year postoperative mean MV pressure gradient

| variables | Univariate analysis | | Multivariate analysis | |
| --- | --- | --- | --- | --- |
|  | Mean ratio (95% CI) | p-value | Mean ratio (95% CI) | p-value |
| Wrapping suture | 0.709 (0.654-0.770) | <0.001 | 0.802 (0.728-0.884) | <0.001 |
| Age (years) | 0.999 (0.996-1.00) | 0.411 |  |  |
| Loop technique or chordal reconstruction | 0.905 (0.827-0.990) | 0.030 | 1.10 (1.00-1.20) | 0.042 |
| Resection and suture | 1.13 (0.893-1.44) | 0.303 |  |  |
| Leaflet plication | 1.07 (0.989-1.16) | 0.093 |  |  |
| Height reduction | 0.880 (0.756-1.02) | 0.099 |  |  |
| Posterior leaflet extension or patch reconstruction | 1.32 (1.05-1.65) | 0.016 | 1.26 (1.03-1.55) | 0.027 |
| Rigid or semirigid ring | 1.27 (1.12-1.44) | <0.001 | 1.17 (1.03-1.33) | 0.015 |
| Full ring | 1.11 (0.991-1.25) | 0.072 |  |  |
| Ring size | 0.941 (0.927-0.955) | <0.001 | 0.948 (0.933-0.964) | <0.001 |
